# *Argonaute 2* in Neurons is essential for healthy Blood Brain Barrier Formation

**DOI:** 10.1002/alz.089858

**Published:** 2025-01-03

**Authors:** Chandan Sona, Yu‐Te Yeh, Adhideb Ghosh, Laura C. Hinte, Ferdinand von Meyenn, Xin Yan, Matthew Poy

**Affiliations:** ^1^ Johns Hopkins University, Saint Petersburg USA; ^2^ Johns Hopkins University, Saint Petersburg, FL USA; ^3^ Laboratory of Nutrition and Metabolic Epigenetics, Department of Health Sciences and Technology, ETH Zurich, Schwerzenbach, Switzerland, Zurich Switzerland; ^4^ Max Delbrück Center for Molecular Medicine in the Helmholtz Association, Robert Rössle Strasse 10, Berlin, Germany, Berlin Germany; ^5^ Translational Neurodegeneration Section “Albrecht Kossel”, University Medical Center Rostock, University of Rostock, Rostock, Germany, Rostock Germany

## Abstract

**Background:**

Argonaute2 (*Ago2*) plays an essential role in RISC‐mediated silencing of target mRNAs, which are critical for cellular functions. Argonaute2 Syndrome, also known as *Ago2* Syndrome, is a rare neurological disorder recently discovered in humans. It has significant implications for brain development, yet it remains unstudied to date

**Method:**

To study this effect, we deleted the *Ago2* gene in GABAergic (*Slc32a1* cre) and Glutamatergic (*Slc17a6* cre) mice. We conducted Western blotting and Immunohistochemistry to analyze the defects in knockouts. Additionally, we performed single‐cell experiments and analysis on the knockouts.

**Result:**

Our study highlights the crucial role of *Ago2* in glutamatergic neurons, leading to postnatal lethality due to disruption in the neurovascular unit (NVU) and neuronal positioning in the developing cortex. Surprisingly, the absence of *Ago2* only affected postnatal survival when removed from glutamatergic, not GABAergic neurons. Through single‐cell analysis, we found that loss of *Ago2* in this specific subset led to impaired synapse formation and endothelial cell connections, resulting in neuroinflammation. Dysregulation of the microRNA pathway and upregulation of *Pten* were observed, and de‐repression of *Pten* in *Ago2*‐deficient mice partially rescued the blood brain barrier (BBB) and increased survival

**Conclusion:**

Our study identifies a vital neuronal population where miRNA function is essential for blood‐brain barrier formation. The loss of functional *Ago2* in this neuronal population leads to BBB collapse and neuroinflammation. These observations warrant studies to be conducted in subjects with vascular dementia to investigate if mutations exist in functional *Ago2* that correlate with *Ago2* syndrome.

